# Federated learning in medicine: facilitating multi-institutional collaborations without sharing patient data

**DOI:** 10.1038/s41598-020-69250-1

**Published:** 2020-07-28

**Authors:** Micah J. Sheller, Brandon Edwards, G. Anthony Reina, Jason Martin, Sarthak Pati, Aikaterini Kotrotsou, Mikhail Milchenko, Weilin Xu, Daniel Marcus, Rivka R. Colen, Spyridon Bakas

**Affiliations:** 1 Intel Corporation, 2200 Mission College Blvd., Santa Clara, CA 95052 USA; 2 Center for Biomedical Image Computing and Analytics (CBICA), University of Pennsylvania, Richards Medical Research Laboratories, Floor 7, 3700 Hamilton Walk, Philadelphia, PA 19104 USA; 3 Department of Radiology, Perelman School of Medicine, University of Pennsylvania, Richards Medical Research Laboratories, Floor 7, 3700 Hamilton Walk, Philadelphia, PA 19104 USA; 4 Department of Diagnostic Radiology, The University of Texas MD Anderson Cancer Center, 1400 Pressler St., Houston, TX 77030 USA; 5 Department of Cancer Systems Imaging, The University of Texas MD Anderson Cancer Center, 1881 East Rd, 3SCRB4, Houston, TX 77054 USA; 6 Department of Radiology, Washington University School of Medicine, St. Louis, MO 63110 USA; 7 Hillman Cancer Center, University of Pittsburgh Medical Center, Pittsburgh, PA 15232 USA; 8 Department of Radiology, University of Pittsburgh, Pittsburgh, PA 15213 USA; 9 Department of Pathology and Laboratory Medicine, Perelman School of Medicine, University of Pennsylvania, Richards Medical Research Laboratories, Floor 7, 3700 Hamilton Walk, Philadelphia, PA 19104 USA

**Keywords:** Computational science, Biomedical engineering, Scientific data, Brain imaging, Medical imaging, Health care, Cancer, CNS cancer

## Abstract

Several studies underscore the potential of deep learning in identifying complex patterns, leading to diagnostic and prognostic biomarkers. Identifying sufficiently large and diverse datasets, required for training, is a significant challenge in medicine and can rarely be found in individual institutions. Multi-institutional collaborations based on centrally-shared patient data face privacy and ownership challenges. Federated learning is a novel paradigm for data-private multi-institutional collaborations, where model-learning leverages all available data without sharing data between institutions, by distributing the model-training to the data-owners and aggregating their results. We show that federated learning among 10 institutions results in models reaching 99% of the model quality achieved with centralized data, and evaluate generalizability on data from institutions outside the federation. We further investigate the effects of data distribution across collaborating institutions on model quality and learning patterns, indicating that increased access to data through data private multi-institutional collaborations can benefit model quality more than the errors introduced by the collaborative method. Finally, we compare with other collaborative-learning approaches demonstrating the superiority of federated learning, and discuss practical implementation considerations. Clinical adoption of federated learning is expected to lead to models trained on datasets of unprecedented size, hence have a catalytic impact towards precision/personalized medicine.

## Introduction

Predictive deep learning models show promise in aiding medical diagnosis and treatment, but require very large amounts of diverse data to be broadly effective. A recent study^1^ found that deep learning models overfit on subtle institutional data biases and performed poorly on data from institutions whose data were not seen during training. It was specifically noted how deep learning medical imaging models may rely on confounding factors associated with institutional biases, rather than basing their predictions on the evaluated apparent pathology. Such models may result in good accuracy when tested against held-out data from the same institution, but do not generalize well to external institutions, or even across departments of the same institution. A natural way to increase both data size and diversity is through collaborative learning, where multi-institutional data are used to train a single model.

**Figure 1:**
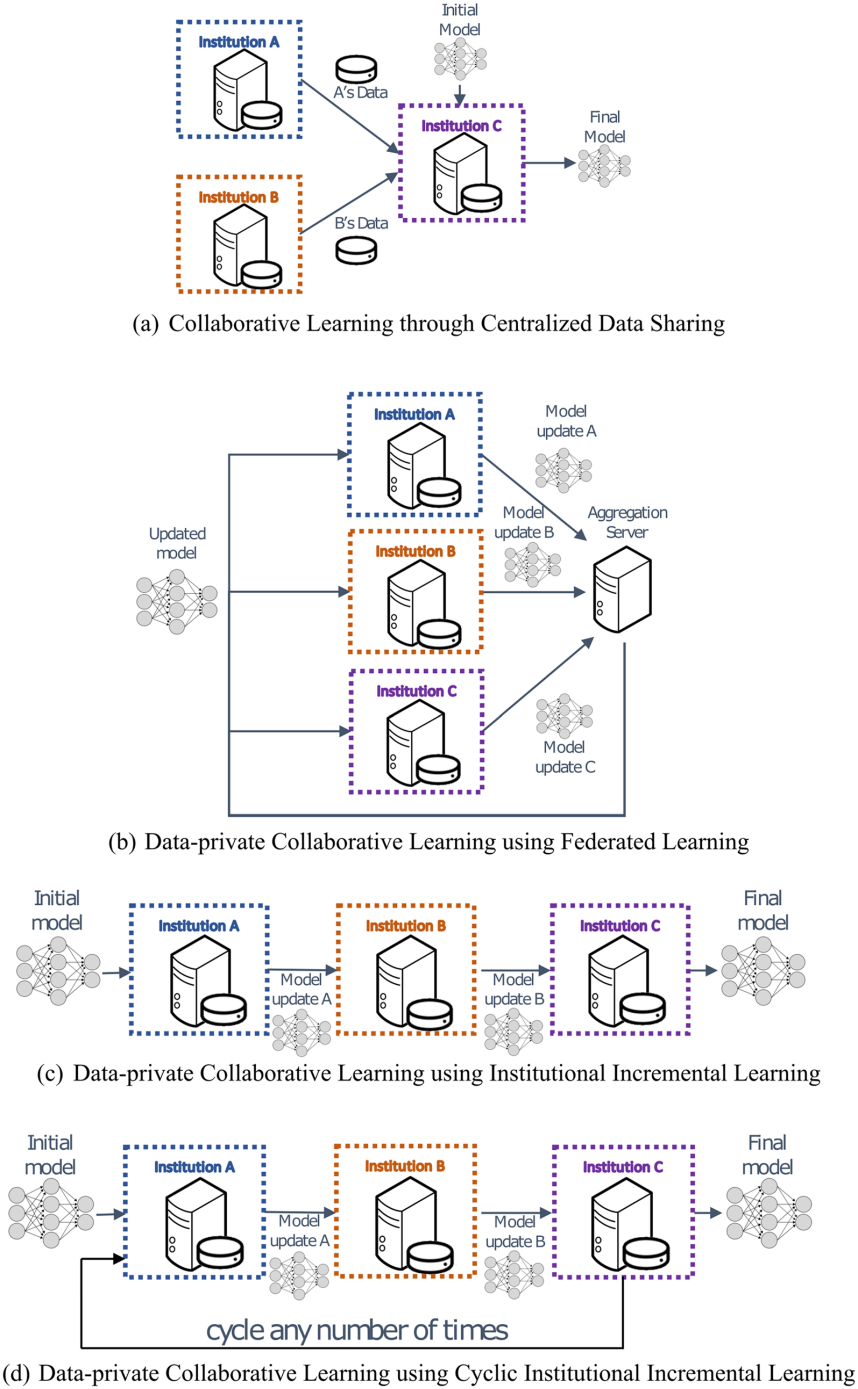
*System architectures of collaborative learning approaches for multi-institutional collaborations.* The current paradigm for multi-institutional collaborations, based on Centralized Data Sharing, is shown in (**a**), whereas in (**b**) we note the proposed paradigm, based on Federated Learning. Panels (**c**) and (**d**) offer schematics for alternative data-private collaborative learning approaches evaluated in this study, namely Institutional Incremental Learning, and Cyclic Institutional Incremental Learning, respectively.

The current paradigm for multi-institutional collaborations in the medical domain requires the collaborating institutions to share patient data to a centralized location for model training (Fig. 1a). Distinct repositories exist for various medical fields, e.g., radiology^2–9^, pathology^10^, and genomics^11^. We refer to this approach as *collaborative data sharing* (*CDS*). However, CDS does not scale well to large numbers of collaborators, especially in international configurations, due to privacy, technical, and data ownership concerns^12,13^. Consequently, knowledge coming from diverse populations worldwide remains distributed across multiple institutions, raising a need to seek alternative approaches. Recent collaborative learning approaches enable training models across institutions without sharing patient data^14,15^. We define such approaches as *data-private collaborative learning*.

*Federated learning* (*FL*)^16^ is a data-private collaborative learning method where multiple collaborators train a machine learning model at the same time (i.e., each on their own data, in parallel) and then send their model updates to a central server to be aggregated into a consensus model (Fig. 1b). The aggregation server then sends the consensus model to all collaborating institutions for use and/or further training. Each iteration of this process, i.e., parallel training, update aggregation, and distribution of new parameters, is called a *federated round*. FL was introduced in 2017 as *federated averaging*^16^, and later applied in training Google’s autocomplete keyboard application^17^.

Chang et al.^14^ explored data-private collaborative learning methods for medical models, where institutions train serially rather than in parallel. We refer to these methods as *institutional incremental learning* (*IIL*—Fig. 1c) and *cyclic institutional incremental learning* (*CIIL*—Fig. 1d). In IIL, each institution trains the model and then passes it to the next institution for training, until all have trained once. CIIL repeats this process, fixing the number of training epochs at each institution and cycling repeatedly through the institutions. The serial training methods of IIL and CIIL can lead to what is technically termed as “catastrophic forgetting”, where the trained model highly favors the data it has most recently seen^18^. The repetitive cycles and limited epochs per institution performed during CIIL enable it to make gradual progress, despite the forgetting, resulting in better models than IIL produces^14^.

The degree to which the institutional datasets used during data-private collaborative learning are independent and identically distributed (IID) can have a large impact on the quality of learning compared to CDS. It can be more effective to iteratively compute model weight updates from batches that mix data across multiple non-IID institutional data sets, rather than iteratively averaging model weight updates, each produced from institutionally dependent batch draws. Zhao et al.^19^ showed that for an image classification task, the performance of their data-private collaborative models dropped by up to 55% depending on how much institutional bias (degree of non-IID) they introduce when sharding (i.e., partitioning) a single dataset into hypothetical institutions. The institutional bias of their hypothetical institutions is created by partitioning according to class label. Medical institution data bias is known to occur^1,20^ caused by demographic differences in served populations, instrumentation bias, and other factors. However, analysis of data-private collaborative methods using artificial data assignments among hypothetical institutions may fail to account for how real-world institutional biases affect the collaborative learning, and the applicability of experimental results to a real-world setting is dependent on how well the experimental datasets model the distributions that will occur in that setting. A natural solution if available, is to experiment with real-world institutional data.

Chang et al.^14^ created institutional datasets by randomly sharding a single set of data into hypothetical institutions (i.e., IID datasets), as well as explored a case where one of those institutions was instead created with an institutional bias (low resolution images, or fewer images with a class label imbalance). Sheller et al.^15^ presented the first evaluation of FL, IIL, and CIIL in the medical domain, over real-world multi-institutional datasets from the International Brain Tumor Segmentation (BraTS) challenge^4–6,21,22^. Importantly, in Sheller et al.^15^ the dataset assignments matched the real-world data distributions, such that all patients from the same hospital were assigned to the same institution. In such real-world configurations, Sheller et al.^15^, confirms that CIIL produces better models than IIL. However, forgetting still occurs during CIIL training, and as a result model quality severely fluctuates. These fluctuations, coupled with the fact that both CIIL and IIL provide no mechanism for validating on the shared dataset during training, resulted in worse models for either CIIL or IIL compared to FL. We have further explored this performance comparison in this present study and we obtained similar results (Figs. 3, 4). Furthermore, it was shown using artificially created institutional data that forgetting can worsen as the number of institutions grows, further reducing the performance of CIIL compared to FL^15^. Li et al.^23^, similarly reconstituted the real-world contributions to the BraTS dataset and compared FL model quality under various training conditions. The primary focus was on the performance impact of differentially private training techniques, which may reduce the risk of training data being reverse engineered from model parameters. Such reverse engineering is one of the many security and privacy concerns that remain for FL, discussed in “Supplementary Information: Security and Privacy”.

Data private collaborative learning introduces additional restrictions to the training process over that of data-sharing (e.g., not shuffling data across participants) as the computational process is not identical (see “Discussion” section). For any given potential collaboration, a crucial question then is whether the increased access to data from data private collaborative learning improves model accuracy more than these restrictions may hamper model accuracy. Here, we take brain cancer as an example, and perform a quantitative evaluation of data-private collaborative learning on the task of distinguishing healthy brain tissue from cancerous tissue, by virtue of their radiographic appearance on clinically-acquired magnetic resonance imaging (MRI). We reconstitute the original 10 institutional contributions to the data of the largest manually-annotated publicly-available medical imaging dataset (i.e., BraTS^4–6,21,22^), to form the *Original Institution* group for our study such that our dataset assignments match the real-world configuration, and further expand our quantitative evaluation to completely independent data from institutions that did not contribute to this dataset. We quantitatively compare models trained by (1) single institutions, (2) using the data-private collaborative learning methods FL, CIIL, and IIL, and (3) using CDS, by evaluating their performance on both data from institutions within the *Original Institution* group, and data collected at institutions outside of that group. These evaluations reveal that the loss relative to CDS in final model quality for FL is considerably less than the benefits the group’s data brings over single institution training. Though we provide a method for model validation during CIIL that makes it competitive with FL on this group of institutions, the Leave-One-(institution)-Out (LOO) testing on this group highlight the fact that CIIL model quality results are less stable than those of FL (Fig. 4). Our findings also indicate that IIL heavily biases the model toward the last model to train, as is discussed in “Supplementary Information: Hyper-Parameter Selection for IIL and CIIL”. For completeness we discuss practical considerations to be made during implementation, including potential optimizations for training efficiency (see “Supplementary Information: Hyper-Parameter Selection for FL”) and ongoing work on mitigations for remaining security and privacy issues (see “Supplementary Information: Security and Privacy”), and also explore more challenging learning environments—both of which further expose the superiority of FL over CIIL (see “Supplementary Information: Further Challenging Model Quality Across Data-Private Collaborative Methods”). In summary, this present study when compared to our preliminary results^15^ (i.e., the first evaluation of FL, IIL, and CIIL in the medical domain), provides a far more extensive evaluation and highlights the need and ongoing considerations to address security and privacy issues. Specifically, the extensive evaluation is done through use of additional publicly available data from BraTS^4–6,21,22^ and additional private testing data from independent institutions (not included in the BraTS dataset). The additional experiments conducted here attempt to evaluate model generalization under various training schemes comprising (1) single institution training, (2) LOO validation, and importantly (3) exhaustively evaluating performance differences between FL, IIL, and CIIL, by exploring convergence, “model selection”, and the effect of institutional order for IIL and CIIL.

## Results

### Ample and diverse data are needed

**Figure 2:**
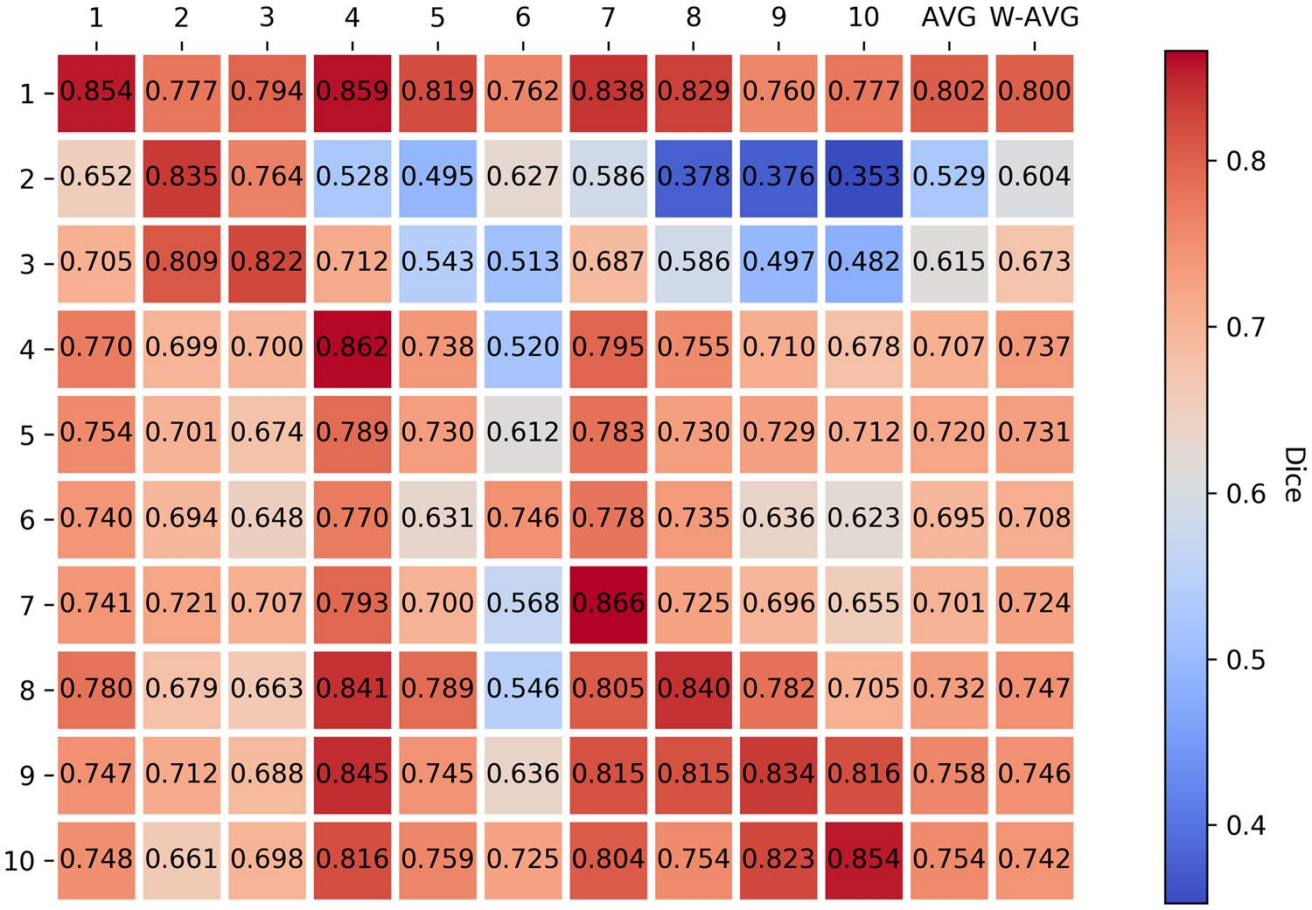
*Single Original Institution Validation Results.* Single institution mean final model qualities (based on the *Dice Similarity Coefficient*^34^) for the *Original Institution group* (y-axis) measured against all single institution held-out validation sets (x-axis) using multiple runs of five-fold *collaborative cross validation*. The Y axis represents models trained on a single institutional dataset, and the X axis represents the validation dataset of each independent institution (Local Validation Dataset). “AVG” indicates the average of each institution mean model performance over all institutions in the group other than itself, “W-AVG” denotes the same, but with a weighted average according to each institution’s contribution to the validation set size. The diagonal entries indicate how well each institution’s final models scored against their own validation set, and they are represented as the Single Institutional Model (SIM) results reported in Fig. 3.

In order to establish the need for more numerous and diverse data at the individual institutions of the *Original Institution* group, we trained single institution models for each institution in the group, and then evaluated each of these models against held-out validation sets from each of the institutions in the group defined prior to model training (Fig. 2).

**Figure 3:**
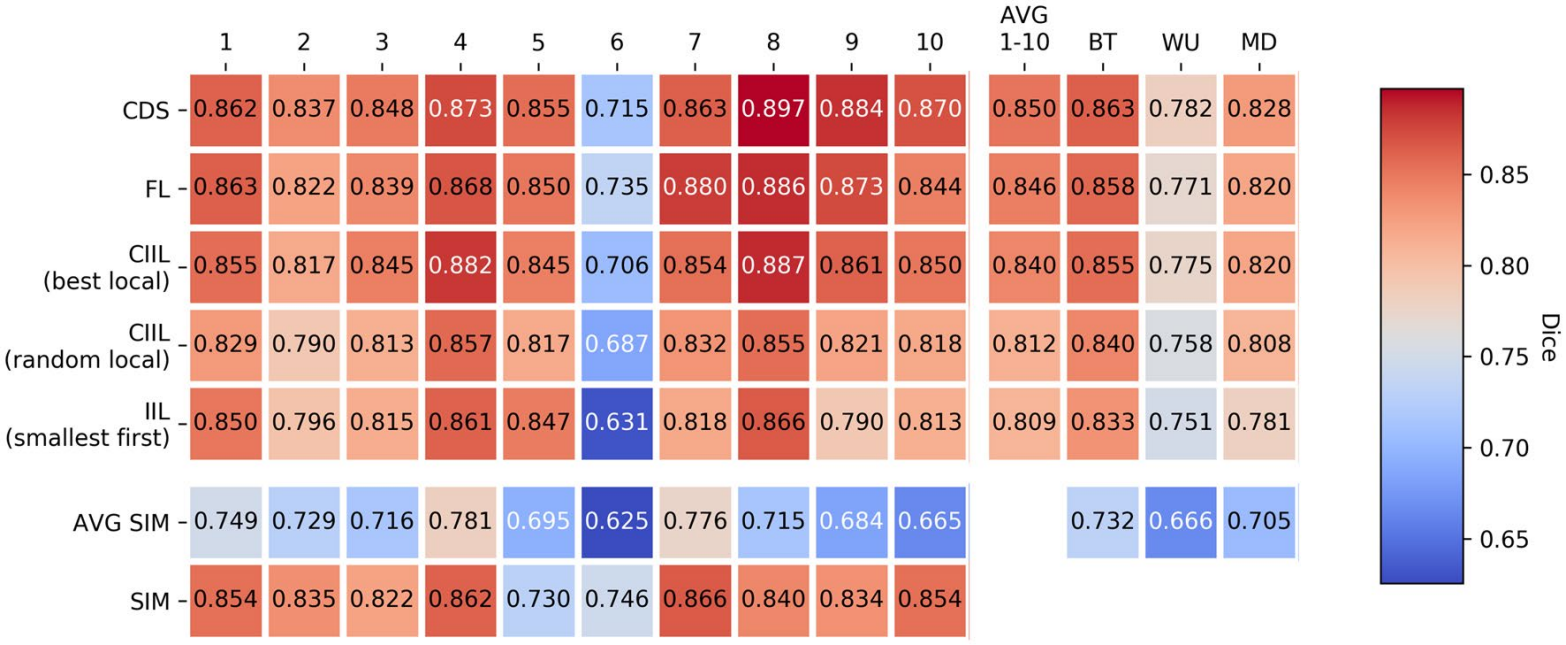
*Model quality results from single institution training, CDS, FL, IIL, and CIIL.* CDS, FL, CIIL mean model *Dice* against the *Original Institution* group single institution held-out validation data over multiple runs of *collaborative cross validation*, as well as the average of single institutional results under the same scheme (AVG SIM). The AVG 1–10 column provides the average performance of each collaboration method across single institution validation sets. For CIIL, ‘best local’ and ‘random local’ are two methods we introduce for final model selection during CIIL (More details are given in the “Methods: Final Model Selection” section ). Note that the color scale here differs from that used in Fig. 2.

We note that institutional models perform much lower against data from the other institutions of the group, showing that more ample and diverse data are indeed needed by each institution to train more generalizable models—a fact that is also supported by the results in our next finding. Note also that institution 1 has by far the best generalization performance. Institution 1 also holds the most data in the group (see “Methods: Data” section for more details). The poorest model generalization performances are shown on institutions 2, 3 and 6, which have the smallest data contributions of the group.

### Collaborative learning is superior

We evaluate the benefits of collaborative learning with respect to improving both scores on an institution’s own data, and the generalization performance to data from unseen institutions. In both evaluations, we compare models trained only on data from each single institution against models trained collaboratively using CDS and FL. To evaluate the first goal, we compare models over the single institutions’ local held-out validation sets (For more details see “ Methods: Data” section) to determine whether a given institution can improve performance on its own data by collaborating. To evaluate the second goal, we compare models over data from institutions that did not participate in the *Original Institution* group.

Figure 3 shows the average (over experimental runs) of the model quality (*Dice*) results for single institution, CDS, and FL models, measured against the local (single institution) validation sets. Notably, averaging over institutions, the CDS model performance is 3.17% greater than the single institution models on their own validation data, and for FL the increase is 2.63% (percent improvements are shown in Table S1).

Table 1 includes the average mean and standard deviation of test *Dice* results of models trained using CDS, FL, and data of each single institution, as well as using a LOO schema, where each institution is held out in turn as the test set. Here, test performance exposes an even broader gap in model quality between the single institution and collaborative models (both CDS and FL).

**Table 1:** Model quality results from single institution training, CDS, and all data-private methods.

Model	BTest	WashU	MDACC	Global val	LOO
Avg single inst	0.732 ± 0.054	0.666 ± 0.045	0.705 ± 0.033	0.733	–
CDS	0.863 ± 0.008	0.782 ± 0.009	0.828 ± 0.007	0.862 ± 0.007	0.84 ± 0.006
FL	0.858 ± 0.004	0.771 ± 0.008	0.82 ± 0.003	0.857 ± 0.007	0.835 ± 0.006
CIIL “best local”	0.855 ± 0.007	0.775 ± 0.013	0.82 ± 0.009	0.853 ± 0.006	0.831 ± 0.012
CIIL “rand. local”	0.84 ± 0.021	0.758 ± 0.021	0.808 ± 0.014	0.824 ± 0.035	0.804 ± 0.031
IIL “smallest first”	0.833 ± 0.006	0.751 ± 0.007	0.781 ± 0.009	0.825 ± 0.007	0.785 ± 0.023
Institution 1	0.826	0.731	0.773	0.824	–
Institution 2	0.614	0.572	0.651	0.628	–
Institution 3	0.700	0.635	0.718	0.702	–
Institution 4	0.751	0.680	0.701	0.747	–
Institution 5	0.753	0.685	0.691	0.733	–
Institution 6	0.708	0.621	0.668	0.709	–
Institution 7	0.721	0.674	0.712	0.732	–
Institution 8	0.755	0.687	0.720	0.755	–
Institution 9	0.745	0.691	0.715	0.755	–
Institution 10	0.751	0.687	0.700	0.745	–

Mean ± standard deviation of *Dice* for all collaboration methods on the *Original Institution* group under multiple runs of *collaborative cross validation*, as well as the mean of single institutional results under the same scheme. The LOO results are a weighted average over institutional LOO tests, weighted by test institution contribution. The ‘–’ entries in the LOO column indicate single-institution tests, where the LOO method did not apply.

We see the benefits of collaboration for the ten institutions in our study, both in terms of their own data and in terms of external test data, as rooted in the inherent diversity that can come from data collection across multiple institutions. Collaborative training across multiple institutions is a natural means by which to address the need that deep learning models have for ample and diverse data.

### FL performs comparably to data-sharing

Table 1 shows the mean model test *Dice* of models trained using FL on the *Original Institution* group. Specifically, for the LOO results, the collaborative method is carried out with one institution held-out from training, the held-out data to be used as the test set for the resulting models. The ‘LOO Test’ results reported in Table 1 are the weighted average over institutional LOO tests, weighted by the test institution contribution. These LOO results differentiate FL from IIL and CIIL, and do not include single institution models as these are not trained using data from multiple institutions. The per-institution LOO results can be found in the Supplementary Information Section “Extended Data”. Notably FL performs within 1% *Dice* of CDS on the three test sets, as well as for the LOO tests (on average).

**Figure 4:**
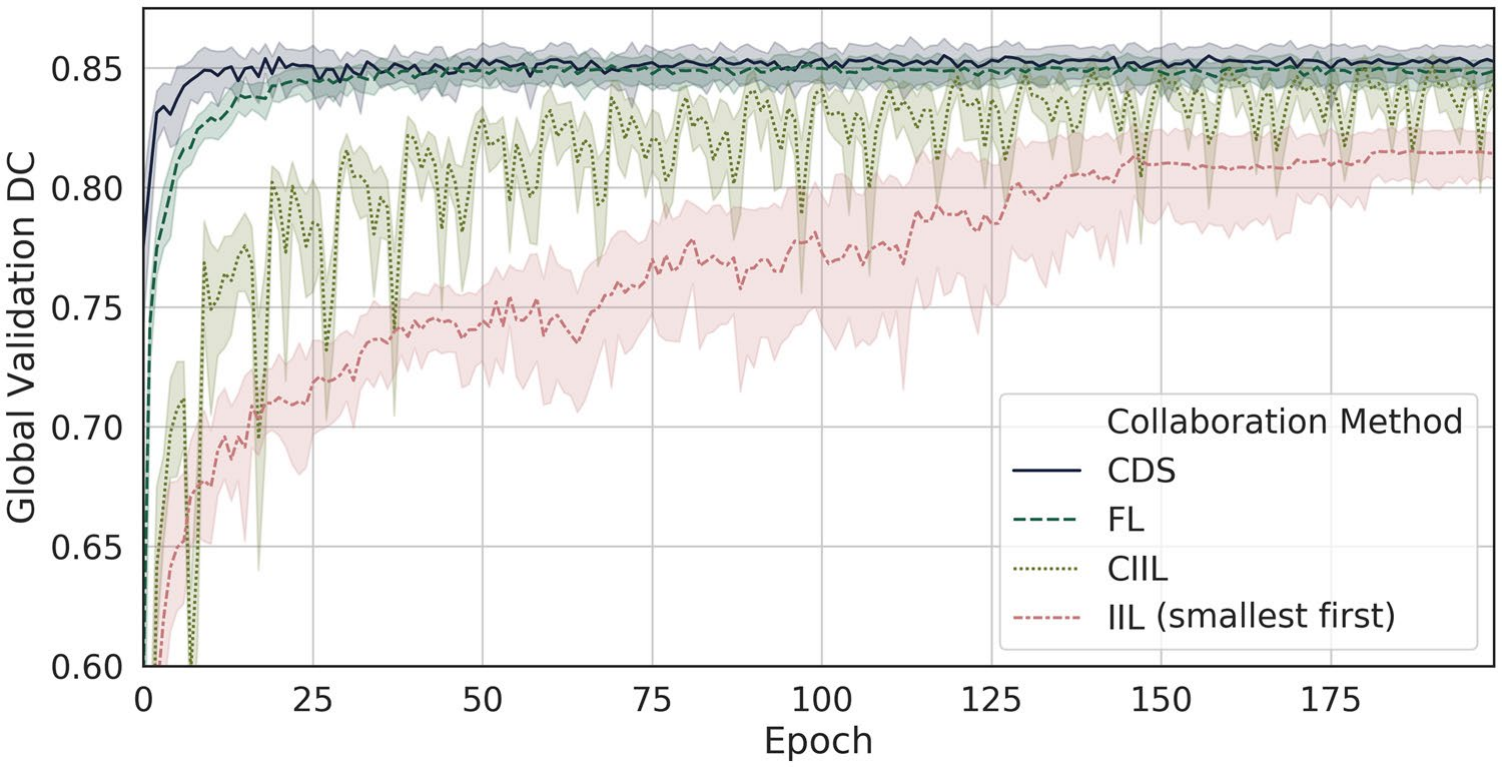
*Learning curves of collaborative learning methods on Original Institution data.* Mean global validation *Dice* every epoch by collaborative learning method on the *Original Institution* group over multiple runs of *collaborative cross validation*. Confidence intervals are min, max. An epoch for DCS is defined as a single training pass over all of the centralized data. An epoch for FL is defined as a parallel training pass of every institutiuon over their training data, and an epoch during CIIL and IIL is defined as a single insitution training pass over its data.

In order to compare the rates of model improvement, we plotted global validation *Dice* over epoch for all collaborative methods (Fig. 4) and show that FL training converges relatively quickly to the same performance as CDS training. A CDS epoch is defined to be a complete training pass over the shared data, whereas an FL epoch is defined as a parallel pass of all institutions over their own data. Averaging epochs from single institution training updates (i.e., FL) is not as efficient as CDS training, which shuffles the institutions’ datasets together, but both approaches eventually converge to the same performance. Here we measure that FL final models took on average 2.26 × as many epochs to train when compared to CDS final models (with a stopping criterion of 10 epochs with no improvement in the best validation DC observed). We also include learning curves for other data-private collaborative methods (Fig. 4).

### Model learning during FL is more stable than during incremental methods

To identify the superiority of a single data-private collaborative method, we compared the learning performance of FL with IIL and CIIL. FL achieves the best rate of model improvement over epoch of the data-private collaborative learning methods (Fig. 4). In addition, the more erratic nature of the IIL and CIIL curves (compared to both FL and CDS) expose an inefficiency in their training, a topic that we return to in the “Discussion” section. Note that an epoch for IIL and CIIL is defined as a pass of one institution over its training data.

The results in Table 1 also show that FL results in better models on average than every other data-private method on the *Original Institution group*. For CIIL, “best local” and “random local” are two methods we introduce for final model selection (see “Methods: Final Model Selection” section), as the only such methods considered by Chang et al.^14^, was that of keeping the model resulting from the last training cycle of a predetermined number of cycles (see “Discussion” section for more information regarding their final model selection). CIIL “best local” is the best competing data-private method, producing models of quality that is generally less than, but very close to FL (see “Supplementary information: Hyper-Parameter Selection for IIL and CIIL” for results regarding the choice of institutional order used in IIL and CIIL). The experiments on the LOO groups (Table 1) show, however, that CIIL “best local” can be less stable, as the standard deviation of model quality is twice or more that of both CDS and FL. See “Supplementary Information: Further Challenging Model Quality Across Data-Private Collaborative Methods”, for experiments on a more challenging hypothetical group of institutions for which CIIL “best local” final model quality mean drops further below that of FL, with an even larger standard deviation relative to FL.

## Discussion

This study shows that data-private collaborative learning approaches, and particularly FL, can achieve the full learning capacity of the data while obviating the need to share patient data, and hence facilitate large-scale multi-institutional collaborations, while overcoming technical and data ownership concerns and assisting towards meeting the requirements of data protection regulations (e.g., the European General Data Protection Regulation (GDPR)^24^, and the Health Insurance Portability and Accountability Act (HIPAA) of the United States)^25^. This finding can potentially pave the way towards shifting the paradigm of multi-institutional collaborations. Model training using FL across multiple authentic institutional datasets performs comparably to model training using CDS (Table 1, Figs. 3, 4). The use of FL over CDS has the immediate advantage of raw data confidentiality, and current technologies can be incorporated into FL to aid in alleviating additional privacy concerns (discussed below). We expect for domains such as medicine, that the development of such solutions will allow for data-private collaborative training over data of unprecedented numbers and diversity. Such collaborations are likely to result in a significant jump in the state of the art performance for these models.

Previous work on CIIL (Chang, et al.^14^) performs final model selection by keeping the last model trained after a predetermined number of cycles. Selecting final models from all locally trained models in this way, makes sense provided models can be consistently validated, and scores shown to be (more or less) non-decreasing. Chang et al.^14^, held out a global validation set for consistent validation, and their results indeed show a non-decreasing trend. We do not see a non-decreasing trend as something one can rely on in general. We think that Chang et al.^14^ was an exceptional case driven by some intrinsic characteristic of their data (such as the IID nature of the data at their hypothetical institutions), and indeed our results confirm that on the contrary a quasi-periodic pattern can be observed. Moreover, CIIL in practice does not allow for anything but local validation. Though we use global validation results to assess the quality of CIIL models, no such set is available to a collaboration in practice without sharing data. Additionally for CIIL, only two of all collaborators ever see any one given model, preventing the aggregation of local validation on the same model that FL uses to obtain global validation results for its model selection process. As a result, we introduce the “random local” and “best local” model selection methods, and consider “random local” as the method closer to Chang et al.^14^ as it requires less communication. We find that “best local” significantly outperforms “random local” in our setting.

Following its performance evaluation, we favor FL over IIL and CIIL as a more principled way to perform multi-institutional data-private collaborative learning. The individual institutional training that occurs during all of FL, IIL, and CIIL is biased in as much as that institution’s data patterns differ from that of the union of data used for CDS training. In the case of FL however, the results of institutional training are aggregated at the end of each round, mitigating this bias. In IIL, a type of aggregation exists as subsequent institutional training blends knowledge into the models it receives from the previous institution, however this aggregation favors institutions that train later in the cycle, and no mitigation exists for bias introduced by the last institution. See “Supplementary Information: Hyper-Parameter Selection for IIL and CIIL” for further evidence of this bias during IIL. CIIL further mitigates individual institutional bias, by limiting the number of epochs each institution trains before passing it forward, and by incorporating repeated cycling in an effort to enhance the type of aggregation that occurs during incremental training. The differences in the time-scale and quality of aggregation that occurs during FL versus IIL and CIIL, create qualitative differences in their training curves (Fig. 4). The short-term performance drops within the IIL training curve in Fig. 4 indicate that when an institution trains, it can significantly reduce previously established performance. Likewise, the CIIL curves clearly show a quasi-periodic pattern formed by re-visiting these performance drops while cycling over the institutions. We see this behavior as indicative of *catastrophic forgetting*^18^. The forgetting is not complete, as is evidenced by the fact that model improvement is still achievable for CIIL over cycles. However, these patterns do expose an inefficiency in the training processes of both IIL and CIIL.

Consistent with the findings of Zech et al.^1^, the CDS models for the *Original Institution* group still appear to suffer from a lack of diverse data, scoring an average of 11% and 5% lower *Dice* on the data from institutions outside of the *Original Institution* group (Table 1, Fig. 3). Though our institutional datasets are somewhat limited to be representative of a standard CDS contribution, we expect that data privacy and ownership concerns prevent near-term multi-institutional CDS collaborations large enough to overcome institutional biases and build models that widely generalize. We believe the data privacy that FL enables will be a catalyst for the formation of much larger collaborations, leveraging data throughout the world, since the data will be retained within their acquired institutions. Hence FL models will substantially benefit by continually learning on new data, compensating for the current relatively inferior performance compared to CDS models. Additionally, some settings may allow for this gap to be further closed, as we further describe in the Supplementary Section “Hyper-Parameter Selection for FL”.

Although the data are not centrally shared in FL, sources of variation across equipment configurations and acquisition protocols require careful consideration. For example, the highest throughput of medical images is produced during standard clinical practice, where the uncontrolled and varying acquisition protocols make such data of limited use and significance in large-scale analytical studies. In contrast, data from more controlled environments (such as clinical trials) are more suitable^26,27^. To appropriately address this issue, common pre-processing routines should be considered and shared that account for harmonization of heterogeneous data (e.g., image resampling, orientation to a standardized atlas), allowing for integration and facilitating easier multi-institutional collaboration for large-scale analytics (see “Methods: Data” for details).

This study focused on the evaluation of data-private collaborative methods in radiographic imaging data. Specifically, following the performance evaluation presented here, the findings of this study support the superiority of FL when compared with IIL and CIIL, particularly on computational models for distinguishing healthy brain tissue from cancer, by virtue of their radiographic appearance. Technically, one can assume that similar results might be expected for other medical deep learning use cases, since generally FL should be able to approach CDS by increasing the rate of synchronization at the cost of network communication overhead. However, we acknowledge that the synchronization used in this study (1 epoch per synchronization, i.e., federated round) may be insufficient for data such as electronic health records^28,29^ and clinical notes, as well as genomics, where more variance might be present across international institutions. Notably, we did not perform hyper-parameter tuning specifically to FL. Further evaluation should be considered for the application and generalizability of data-private collaborative learning in other medical applications, beyond radiographic imaging, including exploration on variations in data sizes, institutional bias, as well as number and sequence of institutions.

While data-private collaborative learning methods keep patient records confidential and allow multi-institutional training without sharing patient data, we caution that privacy risks still exist, since model parameters and the training execution are distributed among the collaborators. Studies have shown that training data may be approximated from the model weights^30,31^. Model parameters necessarily encode information about their training data, which attackers may extract^30^. In FL, CIIL, and IIL the training algorithm is shared with multiple parties, each of which can tamper with some portion of the training. A malicious participant may tamper with training to cause the model to encode more information about others’ training data than is necessary for the model task, improving the attacker’s ability to approximate training data^32^. Thus, while data-private collaborations offer clear privacy advantages over CDS, collaborators must still conduct privacy analyses and consider possible mitigations such as tamper-resistant hardware and proper identity management. See “Supplementary Information: Security and Privacy” for a discussion on such threats and mitigations.

## Methods

### Data

We use the task of distinguishing healthy brain tissue from tissue affected by cancer cells as the case study in evaluation of FL against CDS on a medical imaging task. We used the BraTS 2017 training dataset^4–6,21,22^ to form our institutional training and test datasets. We further formed two additional test sets by utilizing independent additional clinically-acquired brain tumor MRI scans from the University of Texas MD Anderson Cancer Center (MDACC) and Washington University School of Medicine in St. Louis (WashU). The complete BraTS 2017 high grade glioma data were collected from 13 different institutions, and consist of a training set of 210 patient scans, (collected from 10 different institutions), and additional validation and testing sets of 33 and 116 patients, respectively. The WashU and MDACC data consist of 18 and 29 patients, respectively. All these data reflect true clinical practice of radiographically scanning patients diagnosed with gliomas, and consist of multi-modal magnetic resonance imaging (MRI) comprising pre- and post-contrast T1-weighted, T2-weighted, and T2-weighted Fluid Attenuated Inversion Recovery (T2-FLAIR) scans.

The radiographically abnormal regions of each image were annotated and approved by multiple clinical experts at each contributing institution following a pre-defined annotation protocol. The annotated regions included 3 distinct label masks indicating (1) peritumoral edematous/infiltrated tissue, (2) non-enhancing/solid and necrotic/cystic tumor core, and (3) enhancing tumor regions. The raw brain scans were rigidly co-registered to a common anatomical atlas^33^, resampled to an isotropic resolution of 1 mm^3^ to make the size of each scan consisting of 155 axial 2D slice images of 240 × 240 resolution, and skull-stripped. The data were further pre-processed to be made suitable for the specific task of our study, where the affected brain tissue is defined as the union of all three labels described above^4–6,21,22^. Furthermore, following the BraTS annotation protocol we eliminated all but the T2-FLAIR modality.

From the BraTS 2017 training data, we sharded the data across 10 institutions, to match the real-world configuration of the 10 contributing institutions. We call this the *Original Institution* sharding. The resulting patient counts for each of the shards, which we will refer to as institutions 1–10 are given as 88, 22, 34, 12, 8, 4, 8, 14, 15, and 5 patients respectively. Additionally, we formed the *Original Institution* LOO groups from the *Original Institution* group, by variously holding out each one of the ten original institutions. The LOO groups represent additional examples of authentic institutional groups.

Furthermore, for each institution of the collaborative group we hold out a validation set from their data, i.e., *local validation set*. We call the union of *local validation sets* the *global validation* set. These validation sets are used for final model selection as described below.

In order to reduce bias due to local validation set selection, we perform what we call “*collaborative cross validation*”. In *collaborative cross validation*, each institution’s dataset is partitioned into approximately 5 equal folds (indexed partitions), while ensuring that the 155 2D slices coming from a single patient scan end up in the same fold. Every experiment with a different model initialization is performed for five runs, each run using a different fold index to determine the validation fold at every institution. The other four fold indices correspond to the folds that form the training set for every institution during that run. Note that institution 6, holding only 4 patients, will have one empty fold. During CDS and FL, the run for which this fold number is selected is run as usual with no local validation step for institution 6, whereas during IIL, CIIL, and single institution 6 training this run is skipped. All experimental results in this work report average results over multiple instances of *collaborative cross validation*, with each instance using a different model initialization. Note that *collaborative cross validation* defines multiple iterations of coordinated local training and validation splits. As we specify for each experiment we perform, the validation scores reported may come from validating against the global validation set (union of all local validation sets), or from a local validation set belonging to a particular institution.

The BraTS 2017 validation data were combined with 22 cases from the BraTS 2017 test data (moved to the validation set for BraTS 2019) to form one test set for our study, which we call BTEST. (These images are now provided to BraTS 2019–2020 participants during the competition for method development and fine-tuning, and not for ranking purposes. Intel possessed the BraTS 2017–2018 training data having been participants in BraTS 2018 (as the training data were the same for 2017 and 2018). The binarized whole tumor (WT) labels for the BraTS 2017 validation data and the additional 22 BraTS 2017 test cases that were moved to BraTS 2019 validation set, were provided to the lead author Micah Sheller after the conclusion of the BraTS 2018 competition and under a signed Non-Disclosure Agreement. The data were held for calculation, avoiding exposure to a third party, and will be deleted upon publication of this manuscript.) Both WashU and MDACC did not contribute data to the BraTS 2017 training dataset or in the formulated BTEST data, and as such their data is used to test generalization to data from outside institutions. Models resulting from training on each of the *Original Institution LOO* groups are tested against the data owned by the institution held out to form the group.

### Final model selection

Following standard practice, the final model for individual institutional training is taken as the one that achieves the best local validation score over the course of training. For CDS, final model selection can similarly be made using global validation scores. During FL, each institution locally validates any model it receives from the central aggregation server, i.e., at the start of each federated round. These local validation results are then sent to the aggregation server along with the model updates to be aggregated with the other institutional results. In such a way, global validation results can be naturally obtained during FL for final model selection.

Final model selection is harder for IIL and CIIL, than for FL and CDS, as generally no single model is seen by all institutions. Therefore, a complete set of local validation scores cannot be computed within these methods’ natural framework. For CIIL, previous work^14,15^ did not provide any final model selection mechanism. Here, we introduce and explore two final model selection methods that keep close to the minimal communication costs of CIIL. For both these methods, each institution saves the best locally validated model. After the last training cycle, the final model is either randomly selected from one of the locally best models (which we call “random local”) or all locally selected models and corresponding local validation results are passed around in order to select the best local model according to global validation (which we call “best local”). We stress that CIIL “best local” requires more communication between institutions than was originally designed for^14^.

### Model quality metric

To evaluate model quality on a particular test sample, we use a measure (*Dice Similarity Coefficient*^34^*,* also known as *Dice*) in the range [0,1] for the similarity between the model prediction on the test sample features, and the sample’s ground truth mask label. If P and T are the prediction and ground truth masks respectively, *Dice* is defined as:

1 $$Dice = \frac{{2\left\| {P \circ T} \right\|_{1} + 1}}{{\left\| P \right\|_{1} + \left\| T \right\|_{1} + 1}}$$

where $\circ$ is the Hadamard product (component-wise multiplication), and $\left\| {} \right\| _{1}$ is the L1-norm (sum on the absolute values of all components).

For the model training loss, we took the negative log of *Dice,* and explored multiple values for the Laplace smoothing [*s* terms in Eq. (2)]. After algebraically rearranging this loss function, we obtained:

2 $$loss = \log \left( {\left\| P \right\|_{1} + \left\| T \right\|_{1} + s} \right) - \log \left( {2\left\| {P \circ T} \right\|_{1} + s} \right)$$

### The U-net model

For our analysis, we implemented a U-Net topology of a deep Convolutional Neural Network (CNN)^35^, in TensorFlow, and made the source code publicly available at: https://github.com/IntelAI/unet/tree/master/2D (commit: eaeac1fc68aa309feb00d419d1ea3b43b8725773). All experiments use a dropout parameter of 0.2, upsampling set to true, and args.featuremaps set to 32.

### Training hyper-parameters

See “Supplementary Information” for a table summarizing all the hyper-parameters considered in this study. All institutional training in our experiments use mini-batch stochastic optimization and the Adam optimizer^36^, thus require *batch size* and Adam optimizer hyper-parameters^36^ (*adam learning rate*, *adam first moment decay parameter*, and *adam second moment decay parameter*). Additionally, our training loss function requires the smoothing parameter (*Laplace smoothing*) the ‘s’ of Eq. (2) in “Model Quality Metric”. These are the only hyper-parameters required for individual institutional training and CDS, and are shared by FL, IIL and CIIL.

When using the Adam optimizer during FL, each institutional training session results in a distinct final state for Adam’s first and second moments. A natural question arises as to whether it is best to aggregate these moments to be used by every institution in the next training session, or whether it is better to carry forward the optimizer states in some other way. We considered this choice to be an FL-specific hyper-parameter (*optimizer state treatment*). In addition, for FL training one needs to determine how many epochs of training to apply at each institution per round (*epochs per round*), which here we only consider as the same number for all institutions and rounds. One also needs to determine what percentage of institutions to randomly select for participation on each round (*institutions per round*).

Similar to FL, IIL and CIIL also have specific hyper-parameters. No hyper-parameters are associated with the Adam optimizer for institutional training, as for IIL and CIIL we pass the values of the Adam first and second moments along with the model for continued training. Specifically needed for IIL however, is the determination of the number of epochs with no validation improvement (over best so far) before passing the model to the next institution (*patience*), as well as how to order the institutions for the serial training process (*institution order*). For CIIL training one needs to determine how many epochs of training to apply at each institution (*epochs per institution per cycle*), as well as how to order the institutions for each training cycle (*institution order*). We consider only the same *patience* value for all institutions during IIL, the same *institution order* to made during every cycle of CIIL, and the same *epochs per institution per cycle* to be applied at every institution for every cycle of CIIL.

For all institutional training we chose a *batch size* of 64, and used the Adam optimizer with *adam first moment decay parameter* of 0*.*9 and *adam second moment decay parameter* of 0*.*999. In a preliminary experiment, we performed a grid search over the values of the *Laplace smoothing*, and *learning rate* used during CDS training, and found the best cross-validation values to be a *Laplace smoothing* value of 32, and a *learning rate* of 1 × 10^−4^. We subsequently used these institutional training hyper-parameter values for all experiments. See “Supplementary Information: Hyper-Parameter Selection for Institutional Training” for further details regarding institutional training hyper-parameter tuning.

The FL hyper-parameter *epochs per round* and *institutions per round* were set to 1 and 100% respectively in all experiments. Additionally, the FL hyper-parameter *optimizer state treatment* was set to that of aggregating the moments using a weighted average, exactly as the model weights are aggregated during FL. For a discussion of how other values of these hyper-parameters can affect FL training, see “Supplementary Information: Hyper-Parameter Selection for FL”.

All IIL experiments used a *patience* value of 10. For *epochs per institution per cycle* during CIIL, we used 1, as this value produced the best results in previous work^14,15^. For all IIL and CIIL experiments, *institutional order* was taken as increasing order by institution data size as preferable to decreasing order in initial exploration. See “Supplementary Information: Hyper-Paramter Selection for IIL and CIIL” for details of this exploration.

### Experiments

Every experiment in this work was repeated over multiple runs: using multiple random initializations of the U-Net model, with multiple choices for the local validation sets (as discussed in “Data” section).

We first trained models for each institution in the *Original Institution* group using its own training and validation data, training all models to 100 epochs, and evaluating the final model quality *Dice* against all single institution validation sets, the global validation set, as well as BTest, WashU and MDACC test data.

Next, we measure final model quality *Dice* of FL, CIIL “best local”, CIIL “random local”, IIL, and CDS models trained on the *Original Institution* group against the global validation data as well as the BTest, WashU and MDACC test data. Here, all models were trained to 200 epochs.

Finally, we train using CDS, FL, CIIL “best local”, and CIIL “random local” on each of the LOO groups (described in “Data” section). Here all models are trained for a maximum of 200 epochs, stopping early if the best known model by validation did not change over 90 epochs. The quality of these final models was measured as its *Dice* value against the entire training/validation dataset belonging to the institution that was held out to form the group.

## Supplementary information

Supplementary Information 1.

## Abbreviations

CDS: Collaborative data sharing
FL: Federated learning
IIL: Institutional incremental learning
CIIL: Cyclic institutional incremental learning
IID: Independent and identically distributed
BraTS: Brain tumor segmentation
AVG: Average
W-AVG: Weighted average
SIM: Single institutional model
LOO: Leave-one-out
MDACC: University of Texas MD Anderson Cancer Center
WashU: Washington University School of Medicine in St. Louis
CNN: Convolutional neural network
