# Supplementary material for: Federated learning in medicine: facilitating multi-institutional collaborations without sharing patient data
Source: Sci Rep. 2020 Jul 28;10:12598. doi: 10.1038/s41598-020-69250-1 (PMC7387485; doi:10.1038/s41598-020-69250-1)
Supplement: Supplementary file 1 — Supplementary Information 1. [file 41598_2020_69250_MOESM1_ESM.docx]

**Federated Learning in Medicine: Facilitating Multi-Institutional Collaborations Without Sharing Patient Data**

Micah J Sheller^1^, Brandon Edwards^1^, G Anthony Reina^1^, Jason Martin^1^, Sarthak Pati^2,3,[0000-0003-2243-8487]^, Aikaterini Kotrotsou^4,5,[0000−0002−0433−7159]^, Mikhail Milchenko^6^, Weilin Xu^1^, Daniel Marcus^6,[0000−0001−9501−8104]^, Rivka R. Colen^4,5,7,8,[0000−0002−0882−0607]^, Spyridon Bakas^2,3,9,*,[0000−0001−8734−6482]^

1 Intel Corporation, 2200 Mission College Blvd., Santa Clara, CA 95052, USA

2 Center for Biomedical Image Computing and Analytics (CBICA), University of Pennsylvania, Richards Medical Research Laboratories - Floor 7, 3700 Hamilton Walk, Philadelphia, PA 19104, USA

3 Department of Radiology, Perelman School of Medicine, University of Pennsylvania, Richards Medical Research Laboratories - Floor 7, 3700 Hamilton Walk, Philadelphia, PA 19104, USA

4 Department of Diagnostic Radiology, The University of Texas MD Anderson Cancer Center, 1400 Pressler St., Houston TX, 77030, USA

5 Department of Cancer Systems Imaging, The University of Texas MD Anderson Cancer Center, 1881 East Rd, 3SCRB4, Houston, TX, 77054, USA.

6 Department of Radiology, Washington University School of Medicine, St. Louis, MO 63110, USA

7 University of Pittsburgh Medical Center, Hillman Cancer Center, Pittsburgh, PA, 15232, USA.

8 Department of Radiology, University of Pittsburgh, Pittsburgh, PA, 15213, USA

9 Department of Pathology and Laboratory Medicine, Perelman School of Medicine, University of Pennsylvania, Richards Medical Research Laboratories - Floor 7, 3700 Hamilton Walk, Philadelphia, PA 19104, USA

* Corresponding author: sbakas@upenn.edu

# Supplementary Information

**Table S1. Model Quality Results from Single Institution Training*, CDS*, FL, and CIIL Against Single Institution Validation Data.** *Original Institution* group single institution mean model *Dice* against their own validation data over multiple *runs of five-fold collaborative cross validation*, compared to the CDS, FL, CIIL mean model *Dice* against the same validation scheme. For CIIL, ‘best local’ and ‘random local’ are two methods we introduce for final model selection during CIIL (More details are given in the Section “Methods”).

| Local Val. Set | Single Inst.  Model (SIM) | CDS Model  (% above SIM) | FL Model  (% above SIM) | CIIL “best local” Model  (% above SIM) | CIIL “random local” Model  (% above SIM) | IIL “smallest first”  (% above SIM) | IIL “largest first”  (% above SIM) |  |
| --- | --- | --- | --- | --- | --- | --- | --- | --- |
| 1 | 0.854 | 0.862 (+0.9%) | 0.863 (+1.1%) | 0.855 (+0.1%) | 0.829 (-2.9%) | 0.85 (-0.5%) | 0.836 (-2.1%) |  |
| 2 | 0.835 | 0.837 (+0.2%) | 0.822 (-1.6%) | 0.817 (-2.2%) | 0.790 (-5.4%) | 0.796 (-4.7%) | 0.804 (-3.7%) |  |
| 3 | 0.822 | 0.848 (+3.2%) | 0.839 (+2.1%) | 0.845 (+2.8%) | 0.813 (-1.1%) | 0.815 (-0.9%) | 0.802 (-2.4%) |  |
| 4 | 0.862 | 0.873 (+1.3%) | 0.868 (+0.7%) | 0.882 (+2.3%) | 0.857 (-0.6%) | 0.861 (-0.1%) | 0.848 (-1.6%) |  |
| 5 | 0.73 | 0.855 (+17.1%) | 0.85 (+16.4%) | 0.845 (+2.8%) | 0.817 (+11.9%) | 0.847 (+16%) | 0.814 (+11.5%) |  |
| 6 | 0.746 | 0.715 (-4.2%) | 0.735 (-1.5%) | 0.706 (-5.4%) | 0.687 (-7.9%) | 0.631 (-15%) | 0.634 (-15%) |  |
| 7 | 0.866 | 0.863 (-0.3%) | 0.88 (+1.6%) | 0.854 (-1.4%) | 0.832 (-3.9%) | 0.818 (-5.5%) | 0.849 (-2%) |  |
| 8 | 0.84 | 0.897 (+6.8%) | 0.886 (+5.5%) | 0.887 (+5.6%) | 0.855 (+1.8%) | 0.866 (+3.1%) | 0.869 (+3.5%) |  |
| 9 | 0.834 | 0.884 (+6.0%) | 0.873 (+4.7%) | 0.861 (+3.2%) | 0.821 (-1.6%) | 0.79 (-5.3%) | 0.815 (-2.3%) |  |
| 10 | 0.854 | 0.87 (+1.9%) | 0.844 (-1.2%) | 0.850 (-0.5%) | 0.818 (-4.2%) | 0.813 (-4.8%) | 0.788 (-6.7%) |  |
| AVG | 0.8243 | 0.8504 (+3.17%) | 0.846 (+2.63) | 0.8402 (+1.93) | 0.8119 (+1.5%) | 0.809 (-0.6%) | 0.806 (+2.1%) |  |

## Hyper-Parameter Selection for Institutional Training

During training it can occur that the model gets stuck in a local minimum of the loss function at which the model produces zeros for the output of every pixel, i.e., the model predicts that no tumors are present in any image. We refer to this behavior as model collapse. We tried replacing the Dice based training loss with a weighted average of Dice and cross entropy losses, but no values for the weights could be found that reduced the collapse rate.

The collapse was initially observed to be more frequent during data-private collaborative training methods. During data-private collaborative training, batches are drawn with less variation. Going forward however, we restricted our observations to CDS training alone so as to not favor any one particular data-private collaborative method in the solution to the collapse problem.

As we detail in the section “Methods”, we performed an institutional training hyper-parameter search to optimize CDS results. The factors used for this optimization were CDS final model validation quality, and training collapse rate.

Table S2 shows a summary of all the hyper-parameters considered across all training methods in this study.

**Table S2. Summary of Hyper-parameters.**

Name, description, and applicable method for all hyper-parameters considered in this work.

| Hyper-Parameter Name | Applicable Method | Description |
| --- | --- | --- |
| *batch size* | SI, DCS, FL, CIIL, IIL | The batch size to be used for a single step of the optimizer |
| *adam learning rate* | SI, DCS, FL, CIIL, IIL | The learning rate for the Adam optimizer^1^ |
| *adam first moment decay*  *parameter* | SI, DCS, FL, CIIL, IIL | Controls the decay rate for the streaming average of the gradients^1^ |
| *adam second moment decay parameter* | SI, DCS, FL, CIIL, IIL | Controls the decay rate for the streaming average of the square of the gradients^1^ |
| *Laplace smoothing* | SI, DCS, FL, CIIL, IIL | The value of Laplace smoothing to apply in the training loss |
| *epochs per round* | FL | The number of epochs for which each institution trains before sending its update to be aggregated |
| *institutions per round* | FL | The percentage of institutional updates to randomly select for aggregation each round. |
| *Optimizer state treatment* | FL | The choice of how to carry forward the first and second Adam moments when a new aggregated model update is received by an institution for further training |
| *patience* | IIL | The number of epochs of training with no local validation improvement (over best so far) that will trigger a stop to an institutional training session |
| *institution order* | IIL & CIIL | The order in which institutions train |
| *epochs per institution per cycle* | CIIL | The number of epochs for which each institution trains before passing the model |

## Hyper-Parameter Selection for FL

Our experiments all used a value of 1 for epochs per round. Previous work^2^ contains results on training deep learning segmentation models using FL and values of epochs per round 1, 2 and 4. Less than one epoch of training per round could also be considered, replacing epochs per round with partial epochs per round or even batches per round, in order to reach greater rates of synchronization. Changes to the amount of training per round (epochs per round or batches per round) can influence time to convergence, as well as final model qualities. The aggregation step in FL allows for the dissemination of patterns learned at all institutions into the learning steps that are taken at each institution in subsequent training. The benefit of this synchronization depends on the mixture of patterns across institutions, but more frequent synchronization generally brings FL training efficiency (in terms of computation) closer to that of CDS.

The time to convergence includes the time required for the communication exchanges between the institutions and the aggregator. Compression methods could be applied to the model updates to decrease the communication time per round, though such improvements need to be weighed against any adverse effect to final model quality or time to convergence. Recent work^3^ has demonstrated decreased time to convergence when performing FL with compression and less training per round. Institutions per round can also influence time to convergence, and plays a role in differentially private solutions^4^.

We explored three methods for the FL hyper-parameter optimizer state treatment, which determines how to carry forward the first and second moments of the Adam optimizers at each institution when a new round of training begins. The choices considered for optimizer state treatment were:

- RESET: Set both moment values to 0 at the start of the round.
- HOLD: Do not change the values of the moments between rounds (even though the model parameters change with the round update).
- AGG: Aggregate the two moments' values across all institutions, just as with model parameters, and set the moments to these aggregate values at the start of each round (0 for the first round).

Table S3 shows the mean validation model quality results for the different optimizer state treatment choices in FL training on the Original Institution group in a preliminary experiment. For all other experiments where FL is used for the Original Institution group, we use the best performing choice for this experiment (AGG).

**Table S3. Effect of Optimizer State Treatment on Original Institution FL model results.**

Mean ± standard deviation of FL model validation Dice for multiple runs of *collaborative cross validation* on the Original Institution group using different optimizer state treatment methods.

| optimizer state treatment | Validation Dice |
| --- | --- |
| RESET | 0.853 ± 0.008 |
| HOLD | 0.851 ± 0.007 |
| AGG | 0.858 ± 0.005 |

##

## Hyper-Parameter Selection for IIL and CIIL

It was shown for classification tasks^5^ that the value of institutional order can have a significant effect on the quality of model training during IIL, though the effect of the value of institutional order for CIIL training has not been explored. We initially considered two different settings for institutional order for both IIL and CIIL on the Original Institution group: order of increasing data size and order of decreasing data size. Using the Original Institution group, we compared both increasing and decreasing order by size for the hyper-parameter institutional order in CIIL. Model collapse occurred more than twice as often using the decreasing institutional order as it did for the increasing order. In Table S1, we note that institution 3 and institution 2, which sit second and third in the decreasing order respectively, perform very poorly at generalization to validation data at the other institutions. We believe that having these institutions sit so early in the training process influences the model to collapse at the increased rate when compared to CIIL with the increasing order. Similarly, IIL with increasing order was shown to struggle during training on the institutions that sit early in this order (Fig.S1). In addition, the comparative performance on institution 1’s validation data between IIL “smallest first” and IIL “largest first” (Table S1) shows the benefit afforded to the “smallest first” model due to the fact that it’s last training session was on Institution 1 data. The final model quality as measured against the global validation data for the “smallest first” IIL model in Fig.S1 shows how this bias results in a better globally performing model as Institution 1 is the best performing institution with respect to the global data: (Table S1).


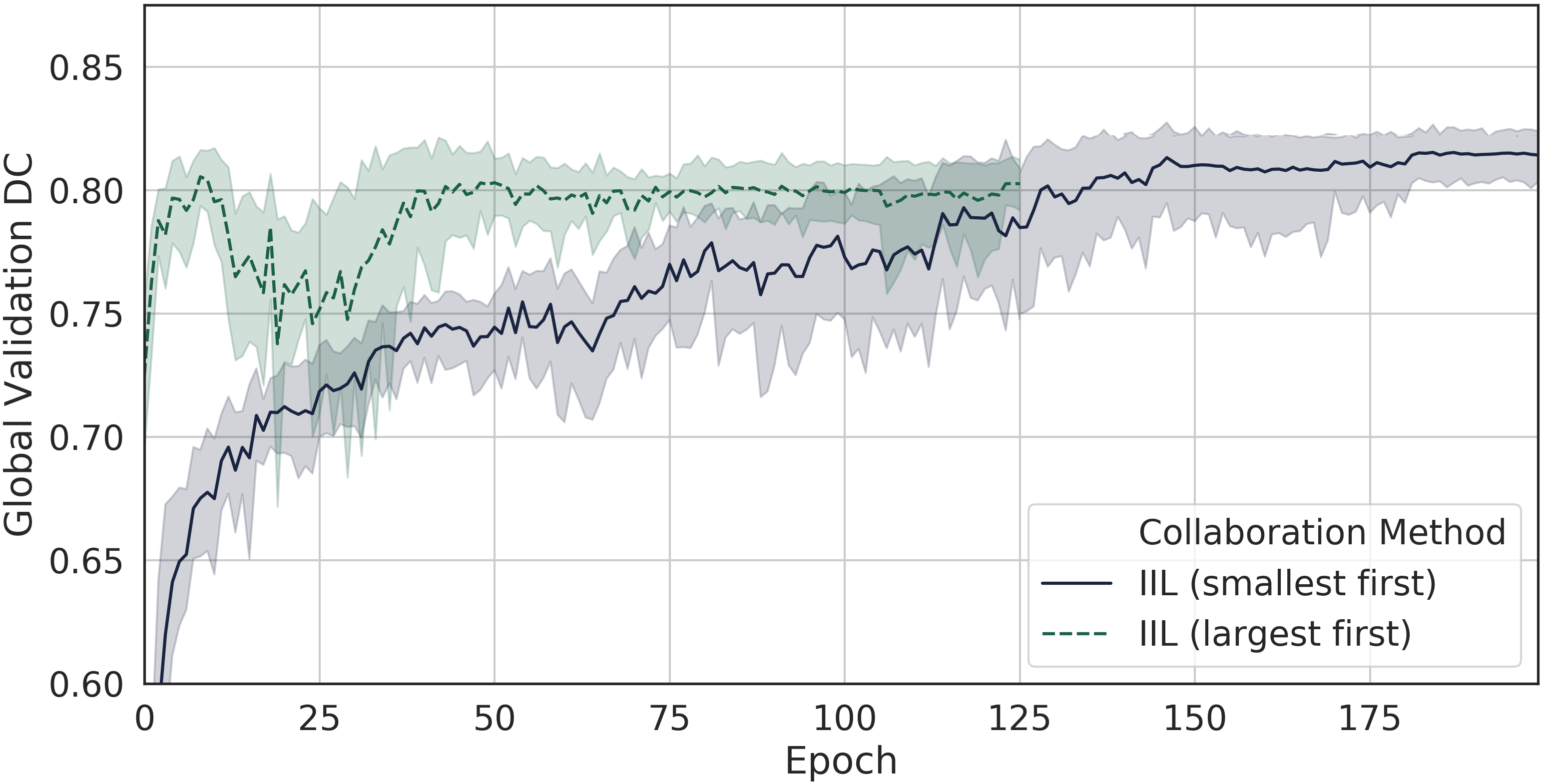


**Figure S1. Effect of Institutional Order on IIL Training on the Original Institution Group.** Mean global validation Dice every epoch for IIL training by institutional order on the Original Institution group over multiple runs of *collaborative cross validation*. Confidence intervals are min, max.

## Further Challenging Model Quality Across Data-Private Collaborative Methods

Despite the performance of CIIL “best local” on the Original Institution group, we have concerns about the volatility in CIIL training due to catastrophic forgetting (Fig.4), as well as the potentially inferior final model selection of CIIL “best local”, relying on per cycle local validation results rather than the per epoch global validation results used in FL. We see both CIIL deficiencies as being exacerbated by large differences across institutions in the data patterns used to learn the global model. However, since this study is focusing on evaluating the use case of distinguishing healthy brain tissue from cancer, by virtue of their radiographic appearance, we did not explore different types of data or other variations that might affect the performance of FL, CIIL, and IIL.

In an attempt to pose a more challenging environment for the data-private collaborative learning methods, we re-sharded the BraTS 2017 training data^6-10^ to form groups of hypothetical institutions by splitting up the largest of the original institutions, and simultaneously increasing the variation in whole tumor sizes represented across institutions. We performed two such re-shardings, one resulting in a group of 12 hypothetical institutions that we call Split 12, and one of 16 hypothetical institutions that we call Split 16. To construct Split 12, we split institution 1 into three institutions, of 30, 29 and 29 patients, with average number of whole tumor pixels per patient of 28502, 63361, and 129610, respectively. To construct Split 16, we split institution 1 into five institutions, and split institutions 2 and 3 each into two institutions. The split of institution 1 resulted in patient counts of 18, 18, 18, 17 and 17, with average number of whole tumor pixels per patient of 22103, 40815, 64073, 92062 and 152958, respectively. The institutions resulting from the split of institution 2 both had 11 patients with an average number of whole tumor pixels per patient of 57946 and 144746, and the institutions resulting from the split of institution 3 both had 17 patients, with an average number of whole tumor pixels per patient of 54751 and 135346.

We train models using the FL, CIIL “best local”, and CIIL “random local” methods on both the Split 12 and Split 16 groups. Final models were compared using their Dice score against the global validation set. Table S4 contains the results combined with the Original Institution results taken from Table 1. The standard deviation of the institutional data label masks’ average whole tumor size are also provided for all of the Original Institution, Split 12 and Split 16 groups.

We see the mean performance of CIIL “best local” can drop relative to FL and CDS, with much greater standard deviation in a more challenging learning environment. For the Split 16 and Split 12 groups of institutions (Table S4), FL models again obtain a mean global validation that is 99% that of the corresponding CDS models for this group (CDS results are the same as for Table 1 (Global Val.), as they do not differ from the Original Institution group). In contrast, CIIL “best local” models obtain a mean global validation that is 98% and 97% that of the corresponding CDS models for the Split 12 and Split 16 groups respectively, with standard deviations of over three times that of CDS and FL in both cases.

**Table S4. FL and CIIL Global Validation Results across Institutional Groups.**

Mean ± standard deviation of FL, CIIL “random local”, and CIIL “best local” model Dice against the global validation set on the Original Institution, Split 12, and Split 16 groups over multiple runs of *five fold collaborative cross-validation* (note these groups all have the same global validation set). Also included is the standard deviation for each group of the per institution average whole tumor mask sizes.

| Institutional Group  (SD of average  whole tumor size) | Original Institution  (0.011) | Split 12  (0.014) | Split 16  (0.017) |
| --- | --- | --- | --- |
| FL Global Val Dice | 0.857 ± 0.007 | 0.856 ± 0.007 | 0.853 ± 0.006 |
| CIIL “best local”  Global Val Dice | 0.853 ± 0.006 | 0.845 ± 0.023 | 0.837 ± 0.025 |
| CIIL “random local”  Global Val Dice | 0.824 ± 0.035 | 0.818 ± 0.036 | 0.796 ± 0.056 |
| CDS  Global Val Dice | 0.862 ±0.007 | 0.862 ±0.007 | 0.862 ±0.007 |

## Security and Privacy

This section specifically focuses on the security and privacy concerns of training a model using collaborative machine learning methods. Some of these threats also apply to inference, but we leave that discussion for future work. We also leave out discussions of common security threats and practices, such as authenticated and encrypted communication, to focus on novel threats to machine learning training.

### Data Private Collaborative Learning Exposes the Model

No patient data is shared by any institution participating in data-private collaborative learning, though model weights are exchanged. Malicious participants may tamper with the model weights with so-called poisoning attacks for malicious reasons, such as biasing a model to perform poorly on a certain patient demographic for the sake of causing a scandal. In some cases, the resulting model may be considered intellectual property (IP). For example, a medical equipment manufacturer may pay some of its customers to use collaborative learning to improve an associated machine learning model, which would ultimately be owned by the manufacturer. In such cases, the learned parameters would likely be considered proprietary. Therefore, we structure our security and privacy analysis of collaborative machine learning around three high-level goals:

1. Prevent inference of private patient data via the model parameters and updates
2. Prevent malicious tampering with the model, e.g. backdoors, model biasing
3. Prevent model IP theft

### Threats Against Models

Protecting patient data goes beyond just preventing direct access, i.e., not sharing data. Machine learning models leak some amount of information about training data, enabling model inversion attacks, wherein attackers partially reconstruct training data^11^. Essentially, attackers use gradient descent to train the input image rather than the weights, and combine this technique with priors on the target to narrow in on realistic reconstructions.

Model poisoning attacks, where the attackers alter the model weights, enable malicious collaborators to bias models (such as over-recommending certain treatments), install backdoors, and generally degrade a model’s performance^12-14^. For example, a collaborator could combine model poisoning and false validation reporting to cause a collaboration to accept a poor final model, while keeping a good version of the model for itself.

Data poisoning attacks attempt to alter model weights through malicious training data. However, such attacks are strictly weaker than model poisoning attacks^15^.

Model poisoning attacks may even cause models to leak more information about their training data than necessary^16^. Thus, attackers may leverage a combination of model-poisoning and model-inversion to reconstruct patient data more effectively than traditional model inversion attacks.

In data-private collaborative training, the model parameters are necessarily shared to the collaborators for training. In the case of proprietary models, this exposes the model to theft via local system attacks such as cold boot attacks, where the attackers read data directly from memory.

### Proper Use of Trusted Execution Environments (TEEs) May Mitigate FL Threats

Trust in an institution can come from trusted hardware, not just legal agreements or other personal relationships. Trusted hardware solutions provide various security properties, depending on the hardware, as well as various compute capabilities. High-end trusted execution environments (TEEs) provide an environment on a computer where computation and memory are hidden from view or influence of even the host operating system, while also providing cryptographic assurances (using a hardware root of trust) of exactly what code is running inside the environment. In the case where a TEE is used for all computations and all data and model information remains encrypted except for within the environment, data-private collaboration can provide a high degree of security and privacy, insofar as the TEE and code are correctly implemented.

High-end TEEs provide cryptographic remote attestations of the code they are running, along with execution integrity to prevent local system operators from subverting the attested code. This provides hardware-based assurances between collaborators that they are running the correct code on the correct platforms, and that those platforms mitigate attacks from local adversaries, i.e., the owner of the platform or a rogue actor with physical access. Execution integrity is critical for any algorithm-based mitigations, otherwise local attackers can subvert the intended algorithms and run malicious code instead.

### Data-Private Collaborative Learning Implies a Different Trust Model than CDS

At a high level, CDS requires trusting one institution fully with both the data and the model. Data-private collaborative learning removes the need to trust anyone with the data, but to various degrees, requires trusting every participant with the model. In institutional collaborations, these trust relationships may be based on the identities of the institutions, i.e., all institutions know and trust each other, including trusting each other to properly secure their systems against attack from outside the collaboration. Such solutions would require an identity framework to verify the ownership of participating machines. These trust relationships may also be based on trusting the security properties of the hardware and software used by the collaborators. Some TEEs provide cryptographic mechanisms for remotely attesting to the hardware capabilities and the software being run. In such cases, collaborators may base their trust primarily in the TEE.

### FL Aggregators are More Capable than FL Collaborators

In FL, a malicious aggregator is a more capable adversary than an FL collaborator. In the context of model poisoning attacks, a malicious FL aggregator can alter the model weights freely. By contrast, malicious FL collaborators can only submit malicious model updates, which the aggregator will combine with other updates to compute the new model. This becomes a contest between the FL aggregator and the malicious participants^15^.

In some cases, the impact of model inversion attacks for reconstruction training data depends on the attacker’s ability to determine which institution contributed the reconstructed data. FL aggregators have access to individual institutional updates which can be used to determine which institution contributed reconstructed data, while FL collaborators only have access to the aggregated model.

### CIIL Collaborators are More Capable than FL Collaborators

Like the FL aggregator, a malicious CIIL collaborator can carry out model poisoning attacks freely before sending the model forward to the next participant, without any need to circumvent the aggregation step. Furthermore, colluding malicious CIIL collaborators can attribute any data reconstructed from model inversion attacks to one of the institutions between them.

### Extended Data

As shown in Fig.S2, we note a missing entry for IIL (largest first) for held-out institution 3. In our experiments, across multiple model weight initializations, that particular sequence of institutions consistently yielded a model that fell into a local minimum of always outputting class 0 for each pixel (no tumor) and was unable to leave that local minimum in the ten epochs of training at each subsequent institution. We have not analyzed this failure enough to conjecture a cause.

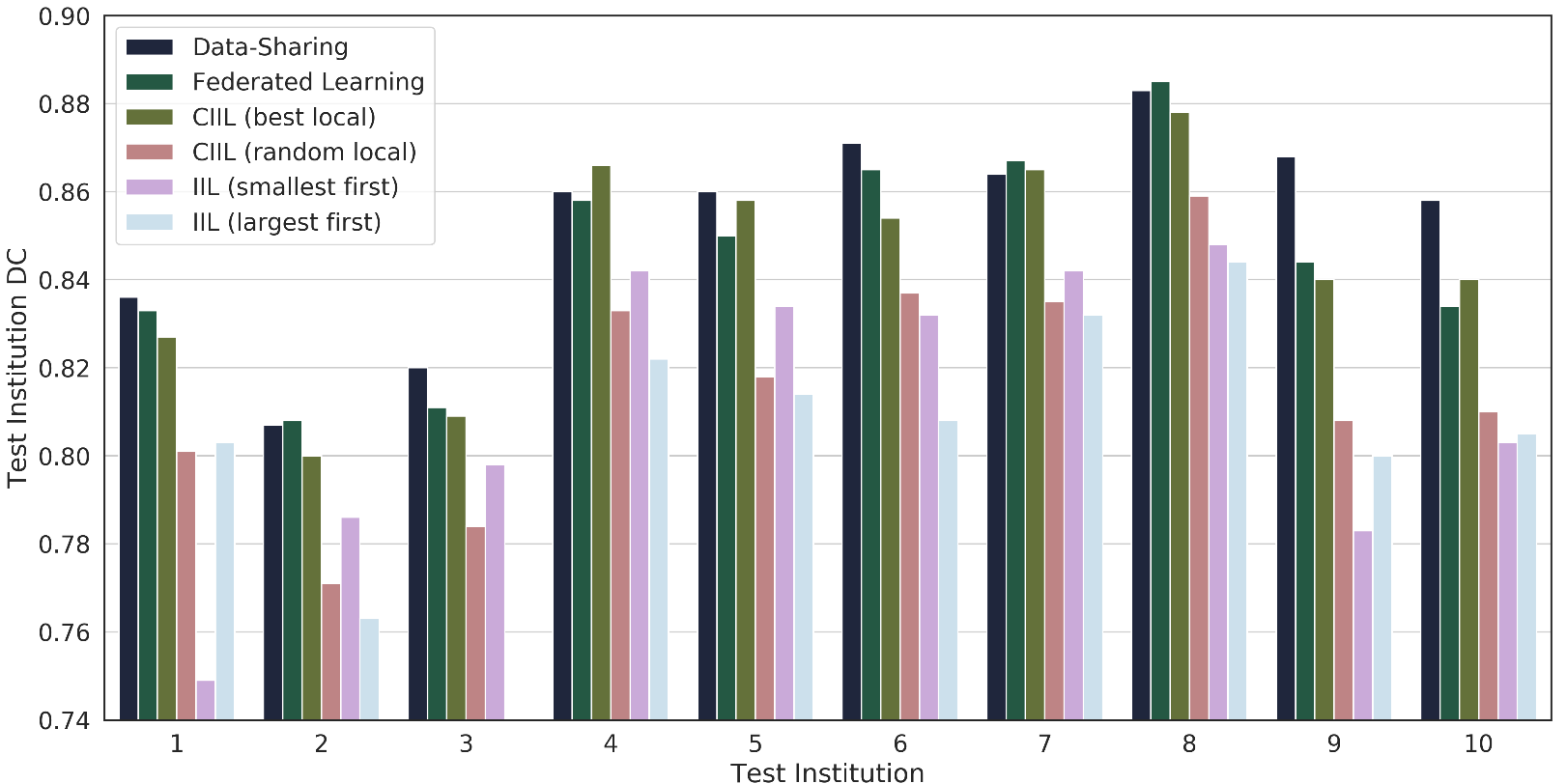


**Figure S2. CDS, FL, IIL, and CIIL *Original Institution LOO* Test Results.** Mean final model test *Dice* by collaborative learning method on each *Original Institution LOO* group over multiple runs of *collaborative cross validation*. The test set being used for a given group is the whole training/validation set of the institution that was held out to form the group.

# References

1 Kingma, D. P. & Ba, J. Adam: a method for stochastic optimization. arXiv:1412.6980 (2014).

2 Sheller, M. J., Reina, G. A., Edwards, B., Martin, J. & Bakas, S. Multi-institutional deep learning modeling without sharing patient data: A feasibility study on brain tumor segmentation. In Brainles 2018 - Springer Lecture Notes in Computer Science 11383, 92–104. https://doi.org/10.1007/978-3-030-11723-8_9 (2018).

3 Sattler, F., Wiedemann, S., Müller, K.-R. & Samek, W. Robust and communication-efficient federated learning from non-iid data. arXiv:1903.02891 (2019).

4 McMahan, H. B., Ramage, D., Talwar, K. & Zhang, L. Learning differentially private recurrent language models. arXiv:1710.06963 (2017).

5 Chang, K. *et al.* Distributed deep learning networks among institutions for medical imaging. *J. Am. Med. Inform. Assoc.* **25**, 945–954. https://doi.org/10.1093/jamia/ocy017 (2018).

6 Menze, B. H. *et al.* The multimodal brain tumor image segmentation benchmark (BRATS). *IEEE Trans. Med. Imaging* **34**, 1993–2024. https://doi.org/10.1109/TMI.2014.2377694 (2015).

7 Bakas, S. *et al.* Advancing the cancer genome atlas glioma MRI collections with expert segmentation labels and radiomic features. *Nat. Sci. Data* **4**, 170117. https://doi.org/10.1038/sdata.2017.117 (2017).

8 Bakas S. *et al.* Identifying the best machine learning algorithms for brain tumor segmentation, progression assessment, and overall survival prediction in the BRATS challenge. arXiv:1811.02629 (2018).

9 Bakas, S. *et al.* Segmentation labels and radiomic features for the pre-operative scans of the TCGA-GBM collection. *The Cancer Imaging Archive.* https://doi.org/10.7937/K9/TCIA.2017.KLXWJJ1Q (2017).

10 Bakas, S. et al. Segmentation labels and radiomic features for the pre-operative scans of the TCGA-LGG collection. *The Cancer Imaging Archive*. https://doi.org/10.7937/K9/TCIA.2017.GJQ7R0EF (2017).

11 Fredrikson, M., Jha, S. & Ristenpart, T. in *Proceedings of the 22nd ACM SIGSAC Conference on Computer and Communications Security* 1322–1333 (ACM, Denver, Colorado, USA, 2015).

12 Carlini, N., Liu, C., Kos, J., Erlingsson, Ú. & Song, D. The secret sharer: measuring unintended neural network memorization and extracting secrets. arXiv:1802.08232 (2018).

13 Wang, Y. & Chaudhuri, K. Data poisoning attacks against online learning. arXiv:1808.08994 (2018).

14 Liao, C., Zhong, H., Squicciarini, A., Zhu, S. & Miller, D. Backdoor embedding in convolutional neural network models via invisible perturbation. arXiv:1808.10307 (2018).

15 Bhagoji, A. N., Chakraborty, S., Mittal, P. & Calo, S. Analyzing federated learning through an adversarial lens. arXiv:1811.12470 (2018).

16 Hitaj, B., Ateniese, G. & Perez-Cruz, F. in *Proceedings of the 2017 ACM SIGSAC Conference on Computer and Communications Security* 603–618 (ACM, Dallas, Texas, USA, 2017).

# Acknowledgements

The authors would like to thank Dr Christos Davatzikos for his insightful comments during writing of this manuscript. Research reported in this publication was partly supported by the National Institutes of Health (NIH) under award numbers NCI:U01CA242871, NINDS:R01NS042645, NCI:U24CA189523 NCI:U24CA204854, and UPMC CCSG P30CA047904. The content of this publication is solely the responsibility of the authors and does not necessarily represent the official views of the NIH.

# Author Contributions:

M.J.S., B.E., and S.B. conceived and designed the complete study. A.K., M.M., D.M., R.R.C., and S.B. provided the data for the study. M.J.S. and B.E. did the data analysis. M.J.S., B.E., and S.B. interpreted the data and wrote the manuscript. G.A.R., J.M., S.P., A.K., M.M., W.X., D.M., and R.R.C. reviewed and edited the manuscript. M.J.S., B.E., G.A.R., S.P., and S.B. created new software used in the study. Each author has approved the submitted version, and has agreed both to be personally accountable for the author's own contributions and to ensure that questions related to the accuracy or integrity of any part of the work, even ones in which the author was not personally involved, are appropriately investigated, resolved, and the resolution documented in the literature.

# Author Information:

Reprints and permissions information is available at www.nature.com/reprints

Correspondence and requests for materials should be addressed to Spyridon Bakas (sbakas@upenn.edu).
